# GrOup based physical Activity for oLder adults (GOAL) randomized controlled trial: study protocol

**DOI:** 10.1186/s12889-015-1909-9

**Published:** 2015-06-27

**Authors:** Mark R. Beauchamp, Samantha M. Harden, Svenja A. Wolf, Ryan E. Rhodes, Yan Liu, William L. Dunlop, Toni Schmader, Andrew W. Sheel, Bruno D. Zumbo, Paul A. Estabrooks

**Affiliations:** School of Kinesiology, University of British Columbia, Vancouver, BC Canada; Department of Human Nutrition, Foods and Exercise, Virginia Tech, Blacksburg, VA USA; Fralin Translational Obesity Research Center, Blacksburg, VA USA; Behavioural Medicine Laboratory, School of Exercise Science, Health and Physical Education, University of Victoria, Victoria, BC Canada; Department of Cell Biology, Harvard Medical School, Harvard University, Boston, MA USA; Department of Psychology, University of California-Riverside, Riverside, CA USA; Department of Psychology, University of British Columbia, Vancouver, BC Canada; Faculty of Education, University of British Columbia, Vancouver, BC Canada; Department of Family Medicine, Carilion Clinic, Roanoke, VA USA

**Keywords:** Physical activity, Older adults, Adherence, Self-categorization theory, Group-dynamics

## Abstract

**Background:**

Physical activity has health benefits across the lifespan, yet only 13 % of Canadian older adults are sufficiently active. Results from a number of observational studies indicate that adults display positive preferences for exercising with others of a similar age and same gender, and that intra-group age- and gender-similarity are associated with elevated exercise adherence. However, research has yet to experimentally examine the extent to which intra-group age- and gender-related similarity affect exercise adherence behaviors.

**Methods/design:**

The GrOup-based physical Activity for oLder adults (GOAL) trial is a three-arm randomized control trial that will examine the efficacy of two different group-based exercise programs for older adults (informed by the tenets of self-categorization theory) in relation to a standard group-based exercise program. Within this manuscript we outline the design and proposed evaluation of the GOAL trial. The first arm is comprised of exercise groups made up of participants of a similar-age and of the same gender; the second arm consists of groups with similar-aged mixed gender participants; the control arm is comprised of mixed-aged mixed gender participants. We aim to compare the adherence rates of participants across conditions, as well as potential moderation effects and mediating mechanisms.

**Discussion:**

Results from this trial will inform intervention designs to improve the exercise adherence behaviors of older adult. At a systems-level, should support be derived for the efficacy of the interventions tested in this trial, changing group composition (i.e., age, gender) represents a feasible program adaptation for physical activity centers.

**Trial registration:**

ClinicalTrials.gov # NCT02023632. Registered December 13, 2013.

## Background

Cardiovascular disease, arthritis, decreased mobility, and obesity represent some of the most prevalent chronic conditions associated with older adults’ physical inactivity [1, 2]. Engaging in 150 min of moderate activity per week is associated with marked improvements for older adults’ reduced risk for cardiovascular disease, functional capacity, and quality of life [3]. Further, those who maintain mobility are more likely to remain in their community of origin for longer [4], which is often associated with higher personal quality of life. In spite of the myriad health benefits associated with physical activity, older adults represent the least active cohort [5, 6], with only 13.1 % of Canadians over the age of 65 (Men = 13.7 %,  Women = 12.6 % ) meeting physical activity recommendations [5].

The current prevalence of physical inactivity has been implicated in the high rate of provincial and territorial government health spending in Canada [7]. Similar to other developed countries (e.g., Australia and United States [8, 9]), 14 % of the Canadian population is 65 years or older with an estimated rise to 25 % by 2036 [10].

From a population health perspective, there are several broad categories of determinants of physical activity that include personal, social, environmental, and policy factors. In the current trial we focus on the *social context* in which physical activity takes place. In particular, the results of meta-analytic reviews suggest that people are more likely to sustain their involvement in physical activity programs if they are provided with the opportunity to exercise with others in social, or group-based, settings rather than on their own [11, 12]. In line with this body of evidence, group-based physical activity programs have been identified as a particularly effective means of promoting sustained physical activity involvement among older adults [13, 14], and also provide an important means of maintaining quality of life and reducing the potential debilitating effects of social isolation that older adults often encounter [15].

Despite the potential for groups to sustain long-term physical activity behaviors, there appears to be an important caveat that comes with exercising with others: If people perceive themselves to be similar to other members of a given group, in terms of salient underlying qualities, this corresponds positively with their attraction to, and level of involvement within, that group [16–20]. If, however, people perceive themselves to be distinctly different from others within their social group, this is likely to undermine their attraction to, and involvement in that group [16–20]. Recent research suggests that across the adult age spectrum people report a positive preference for exercising within groups that are comprised of others their own age [16, 21], and when they participate in such classes they display higher levels of adherence to the group [18]. In a similar regard, people report comparable positive preferences for same-gender rather than mixed-gender physical activity group settings [19]. This preference exists for both males and females although as demonstrated in our recent research the strength of this effect appears slightly stronger for women (*d* = .76) than for men (*d* = .30) [19]. As a complement to these findings, a recent case study of a highly efficacious ‘similar-age-same-gender’ physical activity program for older adults [22] demonstrated noteworthy rates of adherence (with over 45 % of its membership adherent for over 10 years and approximately 70 % adherent for more than 5 years). In light of findings from the above observational studies [16–20] as well as those of the recent case study [22], there is now sufficient evidence to support the development and application of group-based physical activity programs for older adults that incorporate these age- and gender-based considerations, and testing the efficacy of these programs through use of a Randomized Controlled Trial (RCT) design.

### Conceptual framework

Self-categorization theory [23, 24] serves as the conceptual framework for this trial. This theory was developed by Turner and colleagues [23] and built upon its precursor social identity theory [25, 26]. Initially, social identity theory purported that people not only develop a sense of personal (i.e., individualized) identity through reliance upon factors that make them unique, but also possess social identities, based on their membership in social groups [25, 26]. When a social identity is made salient, individuals tend to favor persons who share membership in the applicable social group (i.e., in-group members) over those from other social groups (i.e., out-group members). Although social identity theory included recognition of the fact that social identities will carry implications for both within- and between-group behavior, the predominant focus of this framework centered on between-group (i.e., intergroup) processes [25]. To explicate the cognitive processes by which people categorize themselves and others, and define themselves in terms of membership within different social groups, Turner and his colleagues developed self-categorization theory [23]. This theory focuses to a much greater extent on within-group (i.e., intragroup) processes than social identity theory.

The underlying premise behind self-categorization theory is that people place themselves and others into social categories on the basis of a set of underlying attributes that are particularly salient, and this process of social categorization shapes a range of attitudes, emotions, and behaviors [23]. Specifically, according to self-categorization theory people are generally attracted to others with whom they share membership in a given category (i.e., “birds of a feather flock together”) and repelled by those with whom they do not share category membership [23]. There is a growing body of evidence supporting the notion that the extent to which people self-identify as being similar to, or different from, others within physical activity group contexts, on the basis of social categories such as age and gender influences their attraction to, and level of involvement within, that group [16–20].

### Mediators

In a seminal position paper on developing effective physical activity interventions, Baranowski and colleagues suggested that in order for interventions to be effective in changing behavior, a sound understanding of the ‘key’ psychological determinants (i.e., mediators) of behavior change is required [27]. In the context of this trial, two theoretical mediators – group cohesion and affective attitudes – will be examined to explain the a priori expected relations between involvement in age- and gender-congruent physical activity groups and their adherence behaviors. Group cohesion is defined as “a dynamic process that is reflected in the tendency for a group to stick together and remain united in the pursuit of its instrumental objectives and/or for the satisfaction of member affective needs” (p. 213 [28]), and includes both task and social components. A core theoretical tenet of self-categorization theory corresponds to the similarity-attraction hypothesis, whereby people are more likely to feel attracted to those with whom they perceive themselves to be similar [23]. As such, it stands to reason that when older adults participate in groups comprised of those of the same age and gender, they would be expected to demonstrate greater attraction to the group’s social and task activities, and also perceive the group to be more united (i.e., higher levels of cohesion) [20]. Furthermore, in light of the fact that cohesion has consistently been found to predict improvements in physical activity adherence behavior [11, 12], we would expect that the covariance of the assigned conditions on adherence (at 3 and 6 months) will be explained (mediated) by older adults’ perceptions of task and social cohesion.

A second theoretical mediator that will be examined in this research corresponds to older adults’ affective attitudes. Affective judgments play an important role in many key theories of behavior change and can be defined as the overall pleasure/displeasure and enjoyment expected from a given activity [29]. For example, in the theory of planned behavior, affective judgments are conceptualized through affective attitudes [29], within social cognitive theory they are conceptualized through affective outcome expectations [30], and within self-determination theory they are conceptualized within the intrinsic motivation regulation [31]. In this trial we operationalize the affective attitudes construct as conceptualized within the theory of planned behavior [32]. Affective attitudes correspond to how enjoyable (or unenjoyable) an activity is perceived to be, which contrasts with instrumental attitudes that are concerned with how useful (or useless) that activity is perceived. In a recent meta-analytic review, Rhodes and colleagues [33] demonstrated that affective (rather than instrumental) attitudes significantly predicted physical activity behaviors. That is, people tend to engage in physical activity on the basis of whether they enjoy that activity, and not on the basis of whether it is perceived to carry some future health benefits. This is also consistent with an extensive body of research in social psychology that has found the level of affect (enjoyment) experienced in a given situation is a consistent predictor of the amount of time people choose to spend in that situation [34]. In light of our previous findings that people report a general preference for age-matched [16] and same-gender groups [19] we would expect that the covariance of the assigned conditions on adherence (at 3 and 6 months) will be explained (mediated) by older adults’ affective attitudes (enjoyment) towards those contexts.

### Aims and hypotheses

Drawing from the tenets of self-categorization theory and previous observational research our primary research question was concerned with whether older adults sustain their involvement in physical activity programs (over three and six months) when they participate in groups that are comprised of members of a similar age and same gender, relative to those taking part in similar-age but mixed-gender classes. Both of these conditions will be compared to the adherence of older adults within standard (mixed -age mixed-gender) ‘control’ physical activity groups. Second, we were interested in whether, at the end of a 3-month physical activity program, older adults in SASG (similar-age same-gender) classes will re-enroll in SASG classes over the following 3-month period (6 months in total) to a greater extent when compared to older adults in the SAMG (similar-age mixed-gender) and MAMG (mixed-age mixed-gender) conditions. Our third research question was concerned with whether any group differences among these adherence outcomes can be explained through a mediation model (with cohesion and affective attitudes as target mediators). Our fourth research question was concerned with whether there are gender differences across the primary outcome (physical activity adherence behavior) by assigned condition. Consistent with our previous findings that women demonstrate a slightly stronger preference for same-gender contexts [19] we expected that (in addition to main effects for the SASG context when compared to the mixed-gender settings), the effects for SASG versus the other two conditions (in relation to adherence) would be more pronounced for women. The following hypotheses will be tested:

- *Hypothesis 1.* Older adults in the SASG groups condition will demonstrate improved adherence (i.e., attendance rates) over 3-months and 6-months than older adults in the SAMG condition, who in turn will demonstrate improved adherence to those older adults in a standard (control) group-based exercise condition (MAMG).
- *Hypothesis 2.* A greater proportion of older adults in the SASG condition will re-enroll in the program (after 3 months for an additional 3 months; 6 months total) than those in the SAMG and MAMG groups.
- *Hypothesis 3.* The covariance of the assigned conditions (SASG, SAMG, MAMG) on adherence (at 3 and 6 months) will be explained (mediated) by changes in older adults’ perceptions of group cohesion and affective attitudes (enjoyment).
- *Hypothesis 4.* Gender will moderate the effects of the intervention conditions in relation to adherence and re-enrollment.

In addition to the above outcome assessment analyses, a process evaluation will be conducted to evaluate the procedures embedded within the intervention. This will involve qualitative (interview-based) methodologies. Although no a priori hypotheses will be tested, the process evaluation will provide important insight into both content fidelity (“what is done”) and process fidelity (“how it is done”) of the trial [35].

## Method

### Study design

The GrOup-based physical Activity for oLder adults (GOAL) Trial is a 3-arm randomized controlled trial (RCT) developed in alignment with the tenets of self-categorization theory. The study has been approved by the Behavioral Research Ethics Board at The University of British Columbia, and is registered with ClinicalTrials.gov (# NCT02023632).

The design, conduct, and reporting of this study will adhere to the Consolidated Standards of Reporting Trials (CONSORT) guidelines [36]. The pre-screening process included the completion of the Physical Activity Readiness Questionnaire for Everyone (PARQ+) and Electronic Physical Activity Readiness Medical Examination (ePARmed-X+) [37]. Pre-screening was conducted either by a trained research assistant or the project coordinator (authors SMH and SAW) using a pre-screening script for consistency. If the ePAR-medX+ highlighted that the interested older adult needed physician approval prior to joining the program, the research assistant informed the individual and indicated that subsequent physician approval was required before s/he then could enroll in the study. Following the initial screening process for inclusion/exclusion, informed consent was obtained, and participants were randomized to one of three study arms:

1. Similar-Age Same-Gender (SASG)
2. Similar-Age Mixed-Gender (SAMG)
3. Mixed-Age Mixed-Gender (MAMG)

### Participants

We aimed to recruit 540 older adults that would be randomized across the three conditions. To be eligible participants needed to be 65 years of age or older (targeted recruitment of 50 % female and 50 % male) and not have contraindication which might prevent them from participating in moderate-intensity physical activity. To both effectively manage the trial and avoid unnecessary burden on the respective YMCA centers, the trial was designed to run in two (*N* = 270 in cohort 1, and *N* = 270 in cohort 2) cohorts. The first cohort ran between March and August 2014 and the second cohort is running between March and August 2015. Both cohorts are run within the same March-August window in order to minimize any seasonal effects between cohorts.

### Study interventions

**Fig. 1:**
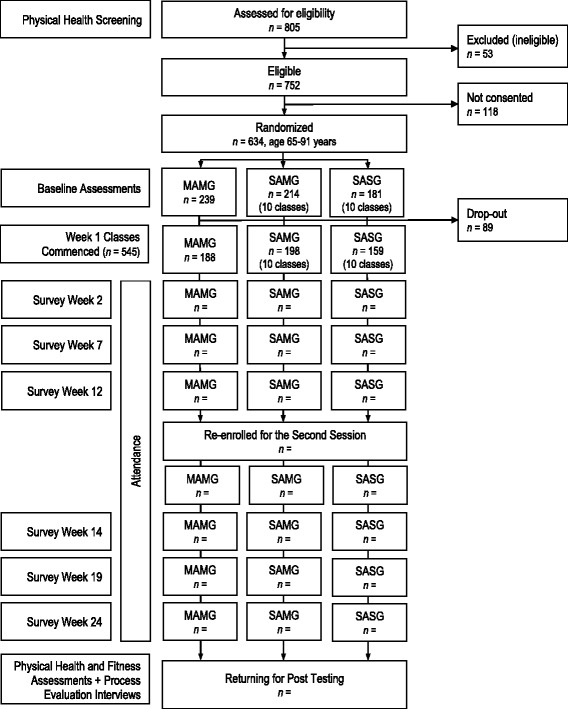
Flow of participants through the trial

The trial is taking place at three different YMCA centers in the Lower Mainland of British Columbia, over 2 years. The group exercise classes take place in 3-month blocks with the opportunity to re-enroll in the same program for another 3 months, thus lasting 6-months in total (see Fig. 1). Each program is run on the basis of classes taking place three days per week, with classes lasting 50–60 min. This dosage (150–180 min/week) is consistent with Canada’s current physical activity guidelines for older adults engaging in ≥ 150 min of moderate-to-vigorous physical activity per week [38], as well as findings from the Canadian Community Health Survey [39] which found that 67 % of seniors who are active three or more times a week are in good health, compared to 36 % who are infrequently active.

The design of the SASG physical activity condition was informed by the tenets of self-categorization theory as well as the results of a recent case study of a highly efficacious physical activity program entitled the *Lively Lads* (a pseudonym) for older adult males [22]. Although the *Lively Lads* program was developed by-seniors-for*-*seniors, it made use of a number of salient theory-driven group dynamics strategies that appear to be implicated in its success. Several of the *Lively Lads* strategies were utilized in the design of the SASG intervention arm of the GOAL Trial. First, one of the core features of this condition is that it designed exclusively for those of a similar age and same gender. Such an environment was reported by Dunlop and Beauchamp [22] to provide opportunities for social connectedness, as well as personal comfort (e.g., reduced likelihood of embarrassment and displays of physical incompetence in front of women). Second, the volunteer exercise class instructors are drawn from the ranks of older adults. Such an approach is consistent with research from the perspective of social cognitive theory, highlighting the value of ‘similar models’ as sources of vicarious efficacy enhancement information and verbal persuasion [40]. From the perspective of program sustainability, volunteer exercise leaders enable the program to keep its costs low (there are no costs involved in paying instructors, as they are volunteers). Consistent with social identity and self-categorization perspectives, the program also makes use of a series of strategies to foster group identity (e.g., providing participants with T-shirts to foster a sense of ‘distinctiveness’). Finally, although a major objective of the SASG intervention condition is to engage in physical activity, an important strategy (informed by both social identity theory and the *Lively Lads* program) is to provide opportunities for the older adults to connect with one another after the classes have ended (e.g., post-workout coffee gatherings). Although the *Lively Lads* program was developed with older adult males, classes are also provided in the GOAL Trial for older adult women at each YMCA site.

The SAMG physical activity condition mirrors the SASG group condition, insofar as the program was restricted to older adults (≥65 years), but was open to older adults from both genders. The same strategies to those used within the SASG condition were also utilized (e.g., T-shirts, opportunities to socialize after the program), with classes also offered on three days per week. Older adults were also recruited to be instructors for the group classes (≥65 years), with both males and females invited to occupy these instructional roles.

The control condition operationalized within the RCT is designed to reflect ‘standard’ group-based exercises that one sees in typical physical activity centers. Specifically, these classes (regular group-based physical activity classes run by the respective YMCAs) are not restricted to participants on the basis of age or gender, and as such older adults in this condition participate in groups comprised of people younger than themselves as well as those of both genders. Specifically, participants randomized to this condition were invited to select one of the standard group-based exercise classes offered by the respective YMCA. Although these classes involve both GOAL Trial participants and regular YMCA members, only the older adults recruited to and consented in the GOAL Trial will be used in the analyses.

#### Program structure

The intervention group classes were developed with the intent of fostering an engaging environment that reflects sound group exercise classes [41]. That is, the research team purchased music playlists that had the appropriate beats per minute (BPM) to align with warm-up/cool-down (120–134 BPM) and moderate intensity physical activity (135–160 BPM). Further, a website was developed to provide instructors (and class participants) with audio, written, and visual (i.e., videos) tutorials for completing exercises that were performed in the sessions. These tutorials made explicit use of both male and female older adult models to demonstrate correct posture and form.

As with the *Lively Lads* program*,* similar-aged volunteer instructors lead the GOAL Trial exercise classes. Instructors were recruited via local media, flyers at the partnering YMCA, and word of mouth. In addition, for the second year of the trial we recruited former participants (from the first cohort) who had expressed an interest to continue as volunteer instructors for the second cohort. Instructors were provided training, through three modules, at one of the three participating YMCAs related to ‘Program Basics and Guidelines’, ‘Tailoring the Program’, and ‘Instructor Mastery’. This training was guided by a Manual of Procedures (available from the first author on request). In the ‘Program Basics and Guidelines’ module, instructors were informed of the study goals and the underlying theoretical principles in lay terminology. They also completed a facility orientation (including First Aid procedures). In the ‘Tailoring the Program’ module, volunteer instructors learned of the different experimental conditions and how to modify the program specific to the SASG or SAMG composition of their group. Finally, instructors had the opportunity to practice leading a group (of the other volunteer instructors) in an exercise class in order to develop their confidence and comfort with the exercises. The duration of volunteer training depended on instructors’ level of certification and previous experience; however, 45 hours of instruction were available.

The instructors were provided with guidance regarding the structure of the program (i.e., to include the warm-up, moderate intensity exercises, and a cool-down). However, each instructor had autonomy on choosing the exercises to be included in each class. Instructors were provided with six unique sequences of recommended exercises with themes of: a full-body, basic class (All Over Burn) as well as classes emphasizing gluteal and back muscles (Spectacular Backsides), abdominal muscles (ABsolutely Intense), upper body (Superhuman Strength), agility and balance moves (Adios Arthritis and Balance Bodies).

#### Outcomes

All pre-screening measures took place in January and February (2014 for cohort 1 and 2015 for cohort 2). Baseline physical health and fitness assessments were conducted in the last week of February, at which point participants also completed questionnaires designed to measure demographic and background variables, physical activity, general health status, as well as a measure of participant personality. In weeks 2, 7, 12, 14, 19, and 24 of the intervention (March to August) participants completed questionnaires that included measures of cohesion, instrumental and affective attitudes, task and self-regulatory self-efficacy, stigma consciousness and psychological flourishing. At week 2 and 14 assessments the questionnaire battery also included measures of intra-group perceptual similarity, commute time, and commute mode. At week 7 and 9, the questionnaire battery also included measures of intra-group communication and group interaction processes. In weeks 12 and 24, the questionnaire battery also included measures of participants’ physical activity behavior outside of their respective YMCA programs. Measures of program adherence were obtained throughout the course of the respective six-month programs. At the end of the respective six-month programs, participants completed the same physical health and fitness assessments as those completed at baseline, at which point the process evaluation interviews were also conducted.

#### Measures

##### Demographic and background measures

Data related to a number of background and demographic variables were collected in relation to participants’ age, sex, country of birth, dwelling arrangements [42], post code (as a measure of socio economic position) [42], and employment/retirement status [43] as well as Canadian Census questions [44] for marital status, ethnicity, level of education, household income. We also collected measures related to participants’ commute time, and mode of transport, to the respective YMCA program.

##### General health status

Data were collected in relation to participants’ smoking status, general health status, previous history of illness, and current use of medication.

##### Physical activity adherence behaviors

Class attendance was objectively measured via reports generated by the use of participants’ YMCA access cards. These data will be used to determine attendance throughout the trial. With regard to the research question concerning the extent to which participants choose to re-enroll after the initial 3-month program has ended, program enrolment records were used (dichotomous: 1 = yes, 0 = no). We collected data at baseline related to participants’ physical activity behavior using Godin’s Leisure Time Exercise Questionnaire (LTEQ) [45], and at weeks 12 and 24 we collected measures related to participants’ physical activity behaviors outside of the program, using procedures described by Wilcox and colleagues [46].

##### Physical health and fitness measures

We collected data related to participants’ height, weight, body composition, blood pressure, functional fitness, and mobility. Specifically height was measured using a standiometer. Both weight (kgs) and body composition (percentage of body fat, as assessed through bio-electrical impedance) were measured using a commercially available portable body composition analyzer (Tanita Model TBF 300 GS, Tanita Manufacturing Co., Tokyo, Japan). Blood pressure was assessed using automatic blood pressure monitors (Life source UA-767 Plus, A&D Medical, USA). These monitors use the oscillometric method to simultaneously provide recordings of systolic (SBP) and diastolic blood pressure (DBP). Participants were required to remain seated for at least 5 min prior to all assessments. Three recordings were made, with an average taken for SBP and DBP. Mean Arterial Pressure (MAP) is calculated according the following equation: MAP = DBP + 1/3(SBP - DBP). Finally, participants completed the functional fitness test for older adults developed by Rikli and Jones [47]. This includes a battery of six tests that assess upper and lower body flexibility and strength as well as aerobic fitness via a 2-min step test. The activities performed during these tests are designed to reflect “the physiologic attributes that support the behavioral functions necessary to perform activities of daily living” (p. 133, [47]).

##### Psychological variables

The primary psychological cognitions targeted in the intervention related to group cohesion and affective attitudes (enjoyment). Class cohesion was assessed using the Physical Activity Group Environment Questionnaire (PAGEQ) [48]. The PAGEQ is a 21-item self-report questionnaire designed to assess four dimensions of cohesion within exercise classes; namely, attraction to the group’s task (ATG-T), and social (ATG-S) activities, as well as perceptions of group integration around the group’s task (GI-T), and social (GI-S) activities. The PAGEQ was developed specifically for older adults taking part in physical activity classes, with scores derived from this instrument found to demonstrate good reliability (α ≥ .72 [48]), factorial validity, and predictive utility. Affective attitudes (enjoyment) towards physical activity were assessed using the procedures described by Rhodes and Matheson [49]. Specifically, a 7-point semantic differential scale was used, with anchors including “Enjoyable—Unenjoyable”, “Pleasant—Unpleasant”, “Interesting—Boring.” Previous research with older adults has found support for both the internal consistency and predictive utility of scores derived from this instrument [49]. In addition to the two primary psychological variables targeted in the intervention (i.e., cohesion and affective attitudes), data were also collected on a secondary set of psychological variables related to participants’ (a) personality [50], (b) task self-efficacy [51], (c) barriers self-efficacy [52], (d) instrumental attitudes [49], (e) stigma consciousness [53], (f) intra-group communication and group interaction processes [54], (g) psychological flourishing [55], and (h) within class perceptual similarity [17, 20].

##### Process evaluation

A process evaluation was conducted to provide insights into both *content fidelity* (“what is done”) and *process fidelity* (“how it is done”) with regard to intervention delivery, as well as the extent to which the intervention meets with the needs of those involved (i.e., older adults) in the program [35]. Without any assessment of intervention fidelity internal validity is potentially compromised [35]. Furthermore, as Plummer and colleagues [56] suggest, process evaluations “can help explain the program’s outcomes and identify ways to improve and/or replicate it. For example, if there are unsatisfactory outcomes, it is important to understand whether this could be due to poor program design, inadequate implementation or special contextual factors.” (p. 500).

In the GOAL Trial, interviews with program participants will enable us to appraise the specific subcomponents of the program and, where appropriate, further modify these for future initiatives [57]. Semi-structured interviews were used that allow us to examine each of the underlying principles of the program (e.g., effects of intra-group age and gender composition, perceptions of class instructor). One of the project coordinators (author SAW) conducted the interviews with participants, and although qualitative data analysis will be overseen by the principal investigator, the coding will be performed by research assistants (i.e., unconnected with the intervention activities) [58].

#### Sample size

We powered our study to detect significant differences in individuals’ physical activity adherence (over 3 and 6 months) in the SASG groups when compared to the SAMG and MAMG standard care control condition. In order to detect a medium effect size *f* = .25 (difference between the SASG and both SAMG and MAMG conditions) based on a 2 (Gender) x 3 (Conditions) ANCOVA with the percentage of classes attended over three and six months specified as dependent variable (while controlling for baseline levels of physical activity) with power (1 - β) set at .80, and alpha set at .05, 211 participants were required across the 3 centers [59]. In order to conduct a logistic regression analysis based on individuals’ re-enrollment across the three group-based programs, based on power at .80, alpha at .05, an anticipated medium effect size (odds ratio = 2.5), and a balanced design, 167 participants were required [59].

To test for mediation through use of a cross-lagged panel model based on a structural equation modeling (SEM) approach, while modeling gender invariance, we drew from three broad criteria. First, based on recommendations provided by MacCallum et al. [60], and using Preacher and Coffman’s [61] R-code for assessing RMSEA (< .05), based on power set at .80, a minimum sample size of 163 was identified as being necessary for conducting the cross-lagged panel model (without considering gender differences). This calculation approximates with recommendations provided by Garver and Mentzer [62] that a ‘critical sample size’ of 200 is required as a general rule of thumb for providing sufficient statistical power for SEM analysis. In addition to these two sets of considerations, in light of the fact that the panel model will include a *multi-group* (males, females) component in order to examine gender invariance, this requires twice the sample size (i.e., *n* = 326, cf. MacCallum et al.; *n* = 400, [60]). Thus, in order to account for an attrition rate as high as 25 % (over the course of the program) a sample size of 540 would be sufficient to examine the mediational model proposed in this trial. In sum, we determined that an overall sample size of 540 older adults across the three arms of the trial would be sufficient for examining each of the study hypotheses.

For the process evaluation component of the trial 15 participants per experimental condition (*n* = 45 in total) will be invited to participate in a semi-structured interview designed to evaluate each of the three experimental conditions (group-based programs) embedded within our trial. It has been suggested that such a sample size is generally sufficient to ensure data saturation with qualitative interview-based data [63].

### Recruitment

Participants were recruited via advertisements placed through the local media, recreation centers, health care centers, hospitals, physician general practices, shopping malls, golf courses, and online interest sites within the Lower Mainland of British Columbia. Eligibility criteria were such that participants must be 65 years of age or older (both males and females) and did not have any contraindications which might prevent that person from participating in moderate-intensity physical activity. We intended to recruit an equal proportion of males and females. Interested persons were asked to call the trial hotline to inquire about program details. The GOAL Trial hotline was used for a pre-screening procedure.

#### Proposed outcome analyses

Preliminary analyses will be conducted to examine whether any patterns of missing data exist (e.g., missing at random, missing completely at random, etc.) for each of the psychosocial variables (cohesion, affective attitudes) using the Missing Value Analysis (MVA; examination of Little’s chi square test) on SPSS Version 20. The data will also be examined for multivariate and univariate outliers, as well as for violations of normality, with the appropriate transformation procedures utilized. Prior to the main analyses, we will also examine invariance in the primary outcome variable (adherence over 3 and 6 months) across the two cohorts (cohort 1 – March to August 2014; cohort 2 – March to August 2015). In light of the fact the two cohorts will be examined at exactly the same time of year, using exactly the same experimental procedures, we would expect invariance in patterns of adherence across the three conditions in each year, thus supporting the pooling of data from both cohorts. Two 2 (gender) x 3 (conditions) ANCOVAs with baseline measures of physical activity (LTEQ scores) entered as a covariate, and adherence to the program over 3-months and 6-months as the dependent variable.

A logistic regression analysis will be conducted to examine the likelihood of older adults randomized to the SASG condition re-enrolling in the same SASG condition after the initial 3-month program, when compared to the re-enrollment of older adults in the SAMG and MAMG conditions. In the logistic regression model, gender will be added as an independent variable, with the regression model explained by $\pi =\frac{1}{1-{e}^{-x}}$ where x = *b0* + *b1G1* + *b2 G2* + *b3 gender* + b4G1*gender + b5G2*gender (where *b0* is the intercept and *b1*-*b5* are the slopes for predictors). For hypotheses 1, 2, and 4, SPSS Version 20 will be used to analyze the data.

Mediation will be tested through use of a multi-group (to examine whether the effects are invariant across gender) cross-lagged panel model using a structural equation modeling (SEM) framework. Cross-lagged panel models are a type of auto-regressive modeling. This approach was proposed by Cole and Maxwell [64] for longitudinal data and will be adapted for the purpose of the present study. This approach has two advantages. Specifically, it allows us to examine the reciprocal relations between the mediators (cohesion and affective attitudes), and the outcome (adherence). In a recent paper we indicated the importance of assessing group cohesion throughout the lifespan of a physical activity group, and not just through a single time-point early-program measure (this has been the most commonly used method of assessing cohesion as a mediator within group dynamics physical activity research [65]). This panel modeling approach, in which all the variables are measured at multiple time points, can rigorously test the prospective relations between predictors (assignment to experimental condition), mediators, and outcomes (i.e., predictors prior to mediators and mediators prior to outcomes). The cross-lagged panel model will be estimated using Mplus 7.2 with a full information maximum likelihood (FIML) estimator used to handle missing data [66]. This procedure will use all available data points for parameter estimation under the assumption that the data are missing at random. FIML estimation tends to produce less biased estimates than deletion or simple missing data imputation techniques (e.g., EM algorithm, regression, listwise/pairwise deletion, mean replacement) even when data are not missing at random [67]. By examining multi-group models, this will allow us to examine whether the structural pathways are invariant across genders.

### Process evaluation analysis

Older adults purposively selected to participate in the process evaluation component of the trial, will be invited to participate in interviews designed to elicit *in-depth* information about the quality of processes embedded within the programs [68]. This component of intervention evaluation will draw from a qualitative *social constructionist* perspective [69] to understand in the older adults’ own words, the beneficial features and any problematic components of the exercise program. Social constructionism is concerned with understanding the manner in which people reflect on and interpret their own and others’ behaviors, and the meanings and values that they ascribe to those interactions. Data collected via the semi-structured interviews will be analyzed through use of inductive content analytic procedures [70], and themes will be identified that correspond to the strengths and limitations of the respective programs.

#### Trial status

In accordance with the proposed time-line, the full program (e.g., recruitment, randomization, baseline testing, six-month physical activity programs, post testing) has been completed for the first cohort of the GOAL Trial. For the second cohort, participants have also been recruited, randomized, and completed baseline measures. Currently, the second cohort of the trial is underway, but (at the time of this protocol manuscript submission) has not yet been completed). No data from either cohort have been subject to any form of data analysis.

## Discussion

Understanding the predictors of physical activity adherence is an important research endeavor within the fields of preventive medicine and health psychology. A growing body of epidemiologic evidence now exists in support of the ongoing involvement in active lifestyles among older adults, and indeed the benefits of regular activity among this population have been well-established [71]. Our proposed study will examine whether a theory-driven, evidence-based intervention has the capacity to support the sustained involvement in physical activity among older adults. The proposed research will also provide the most rigorous test to date of the efficacy of SASG physical activity settings for sustaining the physical activity behaviors of older adults. Although our previous research on exercise preferences [16, 19] as well as the predictive utility of intra-group similarity [16, 18, 20] points to the importance and viability of SASG group contexts for sustaining physical activity adherence behaviors, it should also be noted that these studies utilized non-experimental observational designs. Thus, the causal link between intra-group similarity and exercise adherence has yet to be examined.

In the current study, an experimental (RCT) design is used to examine the efficacy of SASG (and SAMG) settings, the findings of which have the potential to inform the delivery of effective health-enhancing physical activity interventions that are likely to sustain the adherence behaviors of older adults in those programs. If either the SASG or SAMG conditions have significantly higher adherence rates, when compared to the MAMG group, we will have preliminary evidence to support a *small change with substantive impact*. From a knowledge translation perspective, attending to group composition considerations is an easy, sustainable, and low-cost way to influence physical activity behaviors for older adults. This program would easily translate to a variety of physical activity settings including YMCAs, other community-centers, retirement communities, among others. The data collected in this trial have the potential to inform next steps for large-scale implementation and understanding potential mechanisms related to the efficacy of group-based interventions.

There are a few potential limitations to address at the onset. First, the MAMG is unique in that participants can choose any 3-day per week class offered at the YMCA. From a design perspective, there may be a limitation in giving MAMG participants choice in terms of which YMCA course they wished to participate, since participants in the SASG and SAMG conditions were not given any such choice. However, from a pragmatic perspective, we believe that the MAMG group represents the most appropriate type of control condition as it reflects the typical type of exercise class available within community exercise settings. Thus, MAMG provides a strong point of comparison.

There may also be critique of variation related to class leaders. Measures were taken in the extensive training of the volunteer, similar-aged instructors. All volunteer leaders were trained at the same time, through the three modules outlined above. In this way, we sought to increase the likelihood of treatment fidelity across all conditions at each site. However, there may be some inherent differences based on instructor personalities, level of exercise instructor experience, and so forth. To account for these potential differences across conditions and sites, the process evaluation includes queries about perceptions of the class leaders (e.g., leadership and communication style).

## Abbreviations

GOAL: GrOup-based physical Activity for oLder adults
MAMG: Mixed-Age Mixed-Gender
SAMG: Similar-Age Mixed-Gender
SASG: Similar-Age Same-Gender
PAR-Q+: The Physical Activity Readiness Questionnaire for Everyone
ePARmed-X: The Electronic Physical Activity Readiness Medical Examination
